# Quantitative assessment and validation of network inference methods in bioinformatics

**DOI:** 10.3389/fgene.2014.00221

**Published:** 2014-07-16

**Authors:** Benjamin Haibe-Kains, Frank Emmert-Streib

**Affiliations:** ^1^Bioinformatics and Computational Genomics, Princess Margaret Cancer Centre, University Health NetworkToronto, ON, Canada; ^2^Medical Biophysics Department, University of TorontoToronto, ON, Canada; ^3^Computational Biology and Machine Learning Laboratory, Center for Cancer Research and Cell Biology, Queen's University BelfastBelfast, UK

**Keywords:** computational biology, network inference, computational genomics, personalized medicine, bioinformatics, network validation

The last years following the completion of the human genome project (Quackenbush, 2011) have given raise to major breakthroughs in the development of novel biotechnologies, such as next-generation sequencing, that sparked the generation of high-throughput “omics” data. The robustness and the cost-efficiency of these technologies increasing over time enabled the conduction of large screening experiments containing hundreds and even thousands of samples. As a consequence of these “big” biological and biomedical high-throughput datasets, advanced statistical methodology can now be employed requiring such large sample sizes.

This is one reason explaining the recent interest in methods that aim to infer biological networks. These methods offer the opportunity for better understanding the interactions between genomic features and the overall structure and behavior of the underlying networks. In order to foster this research direction we edited a Research Topic entitled “Quantitative Assessment and Validation of Network Inference Methods in Bioinformatics.” This research topic was perceived as relevant and timely by the scientific community and we consequently received 15 contributions from research groups all over the world (Boucher and Jenna, 2013; Chun et al., 2013; de Matos Simoes et al., 2013; Lopes and Bontempi, 2013; Qian and Dougherty, 2013; Schrynemackers et al., 2013; Scott-Boyer et al., 2013; Staiger et al., 2013; Tran et al., 2013; Ho et al., 2014; Horn et al., 2014; Montojo et al., 2014; Olsen et al., 2014; Peng and Schork, 2014; Santra, 2014).

The topics addressed by these contributions can be broadly grouped into the following categories:

- Data integration (Boucher and Jenna, 2013; Chun et al., 2013; Scott-Boyer et al., 2013; Ho et al., 2014; Horn et al., 2014; Olsen et al., 2014; Santra, 2014)
- Network validation (de Matos Simoes et al., 2013; Lopes and Bontempi, 2013; Qian and Dougherty, 2013; Schrynemackers et al., 2013; Montojo et al., 2014; Olsen et al., 2014)
- Network inference (Lopes and Bontempi, 2013; Schrynemackers et al., 2013)
- Time series data (Lopes and Bontempi, 2013)
- Network interpretation (Boucher and Jenna, 2013; Chun et al., 2013; de Matos Simoes et al., 2013; Montojo et al., 2014; Scott-Boyer et al., 2013; Tran et al., 2013)
- Diagnostic applications (Staiger et al., 2013; Peng and Schork, 2014)
- Network modeling (Tran et al., 2013)

First of all, it is important to note that there is still no commonly accepted term to denote 'networks' that are inferred from gene expression data, which the vast majority of the contributed papers used for their inference. Indeed, depending on the context, these networks are called gene regulatory networks (de Matos Simoes et al., 2013; Lopes and Bontempi, 2013; Qian and Dougherty, 2013; Santra, 2014), molecular interaction networks (Horn et al., 2014; Olsen et al., 2014), gene co-expression networks (Scott-Boyer et al., 2013) or biological networks (Schrynemackers et al., 2013). We believe that this plurality denotes the diversity of usages and interpretations of such networks, while it may also reflect the lack of agreement due to the interdisciplinary nature of network inference in Bioinformatics. For the future it would be beneficial to find a common terminology for such networks, because this would certainly enhance the communicability within the community. At the moment, the term 'gene regulatory networks' seems to be the most frequent denotation in use, however, a thorough discussion of this important topic seems indispensable.

The two topics that attracted most interest in the submitted contributions are network validation and data integration. The former is a good reminder that the assessment of inferred networks is not trivial due to two major reasons. First, we still have only partial knowledge about gene regulatory networks even in organisms like *Saccharomyces cerevisiae* (yeast) or *E. coli*, which are considerably simpler than Human. Second, networks are structured objects that means we cannot only assess errors on the global scale for the whole network, but also on intermediate levels down to single interactions and any combination thereof, e.g., motifs or modules (Emmert-Streib and Altay, 2010). In addition, for labeled data enabling the usage of supervised learning methods further issues need to be addressed, as indicated and discussed in the review paper by Schrynemackers et al. (2013).

The integration of different datasets, either of the same or of different types, is certainly a topic that will gain even more attention in the future when more and new high-throughput technologies become available and the access to such datasets is simplified by a policy change of funding agencies making it imperative for grant holders to provide free access to such data. It appears that Bayesian methods (Santra, 2014) provide a natural framework that is particularly suited for such an integration because of its flexibility and widespread acceptance as a fundamental statistical inference paradigm. However, other methods have also been proposed to tackle the challenge of heterogeneous data integration, such as the regression-based framework integrating priors extracted from the biomedical literature and other sources (Olsen et al., 2014). This provides opportunities for comparing novel methodological developments with well-established statistical approaches. We would like to emphasize that networks inferred from the integration of different datasets require a reassessment of their validation for similar reasons as for a supervised learning of gene regulatory networks (Schrynemackers et al., 2013).

For the future, we think that applications of inferred network, e.g., for diagnostic, predictive or therapeutic purposes in medicine will become very important for translational research because of their potential to provide a systems-approach, certainly required to understand complex disorders like cancer. However, until we reach this point more work is needed. For our Research Topic, two contributions have been submitted that are good examples for a better understanding of this problem. In Peng and Schork (2014) the authors found that network centrality measures, which are characterizing the importance of nodes within a gene network that has been constructed from the gene expression patterns, can be used to identify therapeutic targets. In contrast, in Staiger et al. (2013) the authors showed that current composite-feature classification methods considering a network structure, do not outperform simple single-genes classifiers in predicting outcome in breast cancer for prognostic purposes. It is interesting to note that the outcome of both studies allows opposing conclusions. Whereas the results in Peng and Schork (2014) can be seen as an encouragement for further studies employing network-based approaches, the results in Staiger et al. (2013) do not support this. However, by changing the perspective, the study by Staiger et al. (2013) suggests that we do not need to focus on single-gene studies because we can get similar results from network-based approaches. Now, the crucial question is which perspective should we chose? The choice of perspective actually depends on the use of the inferred networks, and therefore the goal of the study. On the one hand, if one is interested in building a predictive model, which does not need to be interpretable (often referred to as “black box” in the literature), then only performance of the inferred model matters; in this case scenario Staiger et al. (2013) showed that, for cancer prognosis, network-based approaches may not be relevant as they do not outperform simpler methods (singe genes). On the other hand, if one is more interested in the biological knowledge that could be extracted from statistical models, network-based approaches are extremely relevant as they are efficient ways to represent complex biological patterns while retaining good predictive ability.

Overall, we believe that, in a translational application, the underlying choice of perspective is of central importance. That means the utility of a network-based approach is expected to depend crucially on the biological question to which such a method should be applied to.

## Conflict of interest statement

The authors declare that the research was conducted in the absence of any commercial or financial relationships that could be construed as a potential conflict of interest.
